# A rare case of hepatosplenic gamma‐delta T‐cell lymphoma and secondary hemophagocytic lymphohistiocytosis

**DOI:** 10.1002/ccr3.1924

**Published:** 2018-11-28

**Authors:** Philip H. Brandt, Leena T. Rahmat, Syed S. Ali

**Affiliations:** ^1^ Department of Hematology and Oncology, D’Amour Center for Cancer Care, Baystate Medical Center University of Massachusetts Springfield Massachusetts; ^2^ Department of Oncology Johns Hopkins University, Sidney Kimmel Comprehensive Cancer Center, Sibley Memorial Hospital Washington District of Columbia

**Keywords:** hemophagocytic lymphohistiocytosis, hepatosplenic T‐cell lymphoma

## Abstract

Hepatosplenic gamma‐delta T‐cell lymphoma with concurrent hemophogocytic lymphohistiocytosis is a rare but well‐recognized clinical scenario, associated with a grim prognosis. Clinicians must be aware of this aggressive type of lymphoma so that a prompt diagnosis can be made with timely initiation of systemic therapy and referral for bone marrow transplant.

## 1 INTRODUCTION

Hepatosplenic T‐cell lymphoma (HSTCL) is a rare type of lymphoma with a poor prognosis. A few cases of HSTCL and hemophogocytic lymphohistiocytosis (HLH) have been described in literature. We report a case of a man with a background of Crohn's disease, which was diagnosed with HSTCL and HLH.

Hepatosplenic gamma‐delta T‐cell lymphoma (HSGDTL) is a rare type of peripheral T‐cell lymphoma and comprises less than 1% of all non‐Hodgkin lymphomas.^1^ It is characterized by an aggressive clinical course.^1^, ^2^ It is included in the World Health Organization (WHO) classification.^3^ HSGDTL is an extranodal lymphoma that most commonly develops in young adults with a median age of 35 years and it tends to have a male predominance.^2^, ^4^ Less than one third of cases arise in patients with a history of immune suppression.^5^, ^6^ One literature review of over 238 cases of HSGDTL reported that 10% of cases occurred in patients with inflammatory bowel disease, who received tumor necrosis factor alpha (TNF‐a) inhibitors and/or thiopurines.^6^

GSGDTL is typified by extranodal infiltration and localization within sinusoids of the liver, sinuses and splenic red pulp.^1^, ^2^, ^4^ Classical presenting symptoms are B symptoms,^1^ and typical physical findings are hepatomegaly and splenomegaly.^1^ Characteristic features also include pancytopenia with bone marrow involvement in 80% of cases.^1^, ^2^ Additional laboratory abnormalities include high serum lactate dehydrogenase, beta‐2 microglobulin level, a transaminitis, and hyperbilirubinemia.^7^

The prognosis of patients with this rare lymphoma is generally poor. Due to its rarity and subsequent paucity of available literature, optimal management remains unknown.^1^, ^2^, ^4^ Response rates and survival rates are poor and current treatment options are limited.^1^, ^2^, ^4^ The median overall survival of patients with HSGDTL is reported to range between 3 and 28 months with the use of several anthracycline‐based chemotherapeutic regimens.^7^, ^8^, ^9^

A number of cases of HSGDTL reported in literature have been associated with hemophagocytic lymphohistiocytosis (HLH), another rare disorder, characterized by an overactivated immune system.^10^ In adults HLH is generally precipitated by an underlying condition.^10^ We report a case of a male with a background of Crohn's disease on chronic immunosuppression, diagnosed with secondary HLH and concurrent HSGDTL.

## 2 CASE PRESENTATION

**Figure 1:**
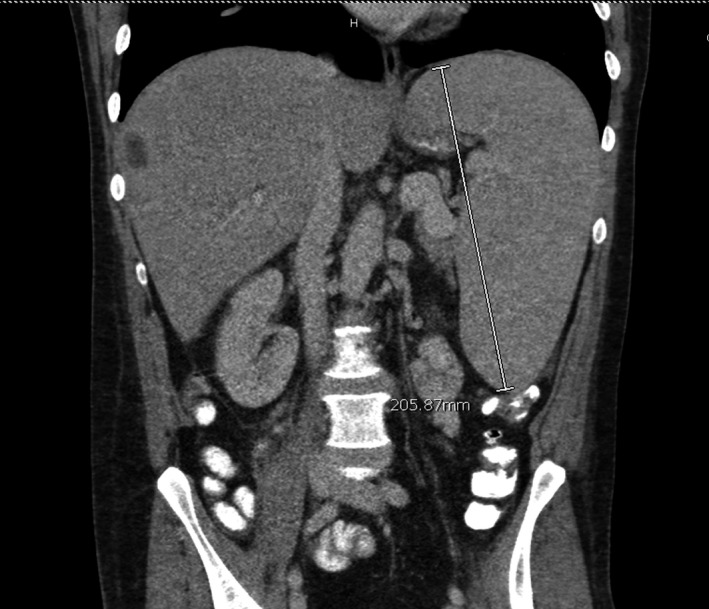
CT scan of the abdomen demonstrates hepatomegaly and splenomegaly

A 55 year‐old Caucasian male with a background of Crohn's disease previously on immunomodulating therapy with azathioprine and adalimumab, presented with subacute fevers, epistaxis, and lethargy. He was noted to have pancytopenia and hepatomegaly, and splenomegaly (21 cm) on CT scan (Figure 1). Physical examination was remarkable for fevers up to 103°F, a diffuse erythematous pruritic rash and splenomegaly without lymphadenopathy. Laboratory revealed a WBC of 1.4 × 10^3^/mm^3^, hemoglobin 7.8 g/dL, platelet 44 × 10^3^/mm3, LDH 460 units/L (3.4‐4.8 units/L), ferritin 936 ng/mL (16‐294 ng/mL), triglycerides 244 mg/dL, and soluble interleukin‐2 receptor 1898 units/mL (<1100 units/mL) and normal liver function tests. An infectious workup was unremarkable.

**Figure 2:**
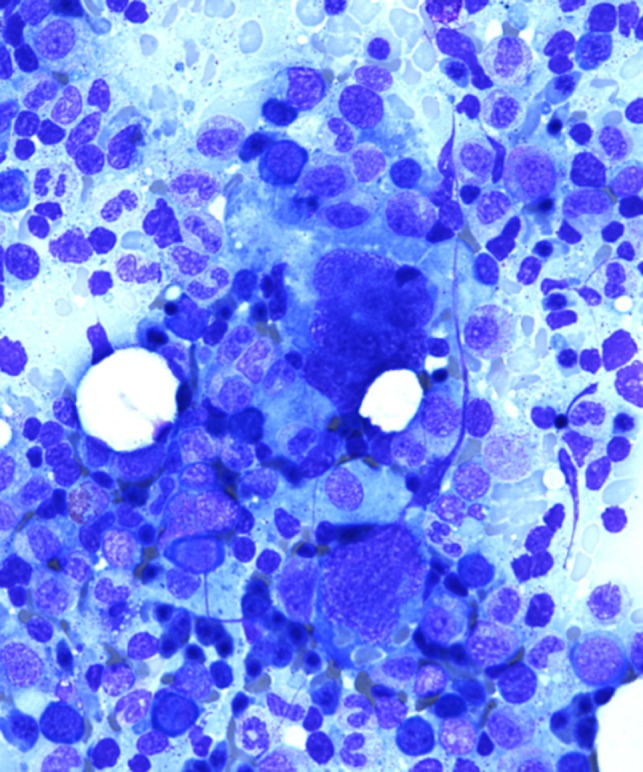
Bone marrow aspirate. High power view. Hypercellular for age (80%). Demonstrates involvement with T‐cell lymphoma and hemophagocytes

**Figure 3:**
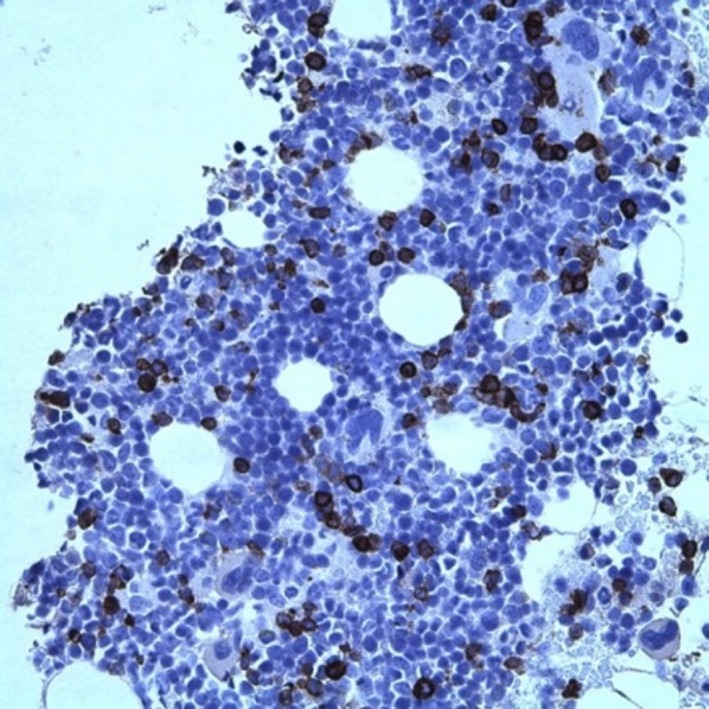
Bone marrow biopsy. Low power view. CD3 immunohistochemical staining consistent with lymphoma cells. Immunophenotyping was positive for CD16, and CD57 and was double negative for CD4 and 8

**Figure 4:**
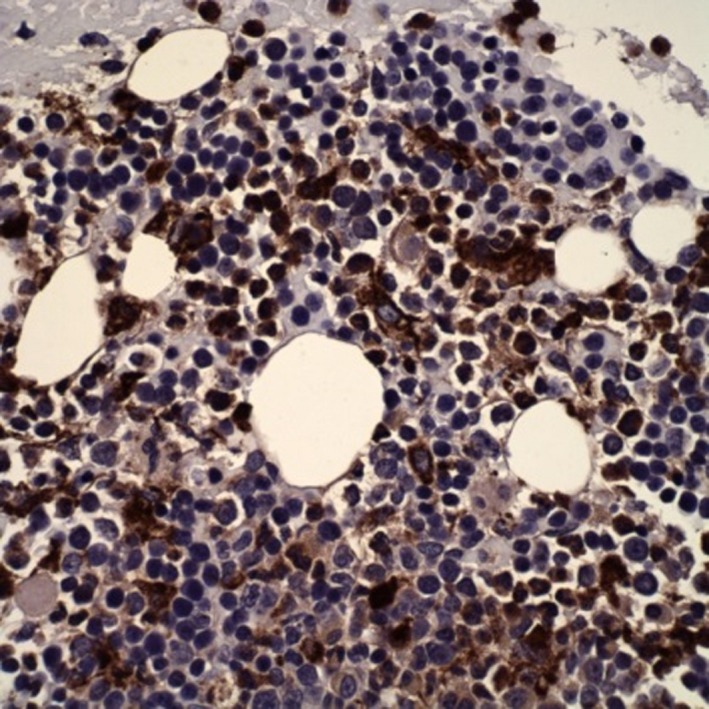
Bone marrow biopsy. The IHC stain was also positive for KP1 (CD68), highlighting the nuclei of engulfed cells within macrophages

A bone marrow aspirate and biopsy revealed T‐cell lymphoma, compromising about 5% of the cellularity as well as a moderate number of hemophagocytes (Figure 2). There is bone marrow involvement in most patients at diagnosis^7^, ^11^ and hemophatocytosis is a recognized but infrequent phenomenon that can develop in patients with HSTCL.^7^ The aspirate demonstrated a hypercellular marrow and normal erythroid number and maturation. Myeloid elements also demonstrated normal maturation and megakaryocytes were present in normal number. Lymphocytes overall did not appear increased however occasional atypical forms were recognizable, although identification of lymphoma cells was difficult. The bone marrow biopsy core demonstrated increased cellularity of 80%. A hypercellular bone marrow is also a typical finding at diagnosis.^7^, ^11^ Lymphoid aggregates were not identified but were appreciated within sinusoidal spaces. Iron stains demonstrated adequate iron stores (3+/6) without pathologic ringed sideroblasts, and reticulin stain showed no increase in reticulin fibrosis. Immunohistochemistry (IHC) stains demonstrated the following: CD20 was negative in neoplastic cells and positive in sparse background small B‐cells. CD3 was positive in neoplastic cells (Figure 3), many within marrow sinusoids, with intermediate nuclei, open chromatin, and a variably prominent nucleolus, overall comprising about 5% of the cellularity. The IHC stain was also positive for KP1 (CD68), highlighting the nuclei of engulfed cells within macrophages (Figure 4). Immunophenotyping by flow cytometry demonstrated the following phenotype: CD3 positive (dim/moderate; dimmer than background small T‐cells), CD2 positive, CD16 positive, and CD56 positive. CD7 was partially positive. CD5, CD4, CD8, and CD57 were all negative. The population comprised about 1% of the cellularity in about 20% of lymphocytes, the remainder predominated by background small T‐cells. HSTCL cells are positive for CD2, CD3, and CD7, and are negative for CD1a, CD5, CD10, TdT, and B‐cell antigens. Approximately 75% of cases express CD56 and the majority of HSGDTL cases are negative for CD57, CD4, and CD8.^4^, ^7^, ^9^, ^12^, ^13^ Cytogenetics was negative for isochromosome 7q; however, the majority of HSTCL cases have an isochromosome 7q [i(7q)] chromosomal abnormality.^3^, ^7^ The lack of isochromosome 7q raised suspicion for other diagnoses including T‐cell large granular lymphocytic (LGL) leukemia and T‐prolymphocytic leukemia. In literature the reported frequency for i[7q] is 25%‐68%.^9^, ^12^, ^13^ The other cytogenetic abnormality that may be found in HSTCL is trisomy 8, reported to have a frequency of 8%‐53%.^9^, ^12^, ^13^ The role of these chromosomal abnormalities is uncertain. It is thought that i[7q] is a driver chromosomal anomaly and that trisomy 8 is a probable secondary event.^14^

**Figure 5:**
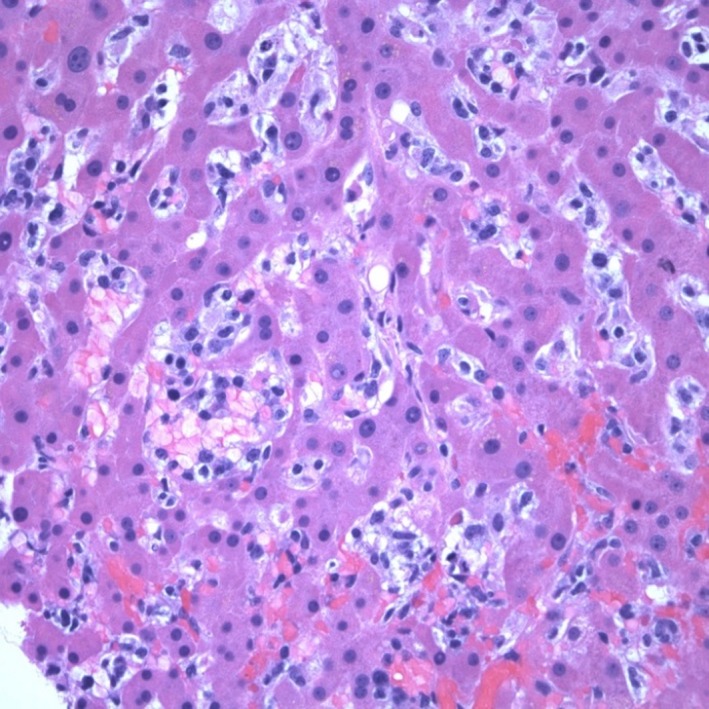
Liver biopsy. High power view. Sinusoidal infiltration of hepatic sinusoids by lymphocytes

The constellation of fevers, splenomegaly, cytopenias, hyperferritinemia, elevated soluble IL‐2, and hemophagocytes bought the diagnosis of HLH. In light of the hepatomegaly, a liver biopsy was pursued which confirmed a diagnosis of HSTCL involvement in the liver (Figure 5). The lymphoma cells infiltrated the liver sinusoids (Figure 5), which is a typical finding with HSGDTL.^1^, ^7^ IHC stains were positive for CD3 and CD56. They were negative for CD20, CD30, granzyme, TdT, and CD1a. There was aberrant loss of CD5. In situ hybridization for Epstein‐Bar virus (EBER) was negative. EBV is commonly negative in HSTCL.^9^

Systemic therapy with chemotherapy was initiated; however, he was refractory to CHOEP (cyclophosphamide, doxorubicin, vincristine, etoposide, and prednisone), ICE (ifosfamide, carboplatin, etoposide), romidepsin, cladribine, and alemtuzumab. Interestingly his cytopenias responded to steroids. He was eventually readmitted with recurrent fevers and worsening hepatosplenomegaly and he died due to progressive disease.

## 3 DISCUSSION

Hepatosplenic T‐cell lymphomas are an uncommon extranodal and systemic malignancy derived from cytotoxic T‐cells usually of gamma‐delta (γδ) T‐cell receptor type.^2^ A lesser number of cases are of alpha‐beta (αβ) type. The peak incidence of HSTCL is in young adults with a median age of 20 years and a male to female ratio of 9:1.^2^ The normal‐cell counterpart for HSTCL is a functionally immature cytotoxic γδ T‐cell of the splenic pool.^15^ The two main types of γδ T‐cell lymphomas recognized by the WHO classification are HSTCL and primary cutaneous γδ T‐cell lymphoma (PCGD‐TCL).^3^ The neoplastic cells tend to localize within sinusoidal regions of the splenic red pulp and in epithelial‐rich tissues.^1^ HLH is a well recognized but rare occurrence that can occur in patients with HSTCL.^1^

The pathogenesis of HSTCL is not fully understood. Literature suggests an association with chronic immunosuppression including patients with Crohn's disease on immunotherapy.^16^, ^17^ One hypothesis is that up‐regulation of the JAK/STAT pathway or mutations in chromatin modifiers such as SETD2 induces a neoplastic clonal proliferation derived from a γδ T‐cell.^1^ Additional transforming events including isochromosome (7q) and trisomy 8 may be involved in the commencement of the full clinical manifestation of HSGDTL.^1^ Furthermore, other mutations reported to be common HSGDTL are STAT3 and STAT5B and mutations in chromatin‐modifying genes including SETD2, ARID1B, and IN080.^18^, ^19^ Less frequent mutations reported in HSTCL are PIK3CD, TP53, KRAS, and EZH2 mutations.^19^ The molecular findings in HSTCL seem to be discrete from other T‐cell lymphomas. Isochromosome (7q) and trisomy 8, which are frequently observed in HSTCL are rarely appreciated in other types of T‐cell lymphoma and leukemia.^20^

The immunophenotype typical of HSTCL is the following: CD2+, CD3+, CD4−, CD8−, CD5−, CD7+, and TCRγδ+.^1^, ^2^, ^3^ At least one NK marker is regularly expressed, including CD56, CD57, or CD16.^1^, ^2^, ^3^ A marginal number of cases are CD8+.^2^ However, not infrequently, there is loss of CD3, CD5, and/or CD7 expression and irregular reactivity for killer immunoglobulin‐like receptors (KIR) with a weak or absent CD94.^9^, ^11^

Long‐term immunosuppression is a strong‐risk factor for the development of HSTCL. Up to 20% of cases of HSTCL develop in patients with long‐termimmune suppression.^1^ The common clinical scenarios are patients with immunodysregulatory disorders, most frequently Crohn’s disease, who are on immunosuppressive agents and post–organ transplantation.^1^ Azathioprine and TNF‐alpha inhibitors such as infliximab have been involved in the pathogenesis of HSTCL.^4^, ^7^ There is a fourfold increased risk of B‐ and T‐cell lymphoma when TNF‐alpha inhibitors are used to treat patients with Crohn’s disease.^17^, ^21^ Rates of γδ T‐cell frequencies are higher in patients with Crohn’s disease.^22^, ^23^ Interestingly, higher levels have been found in patients with active disease.^22^, ^23^ Gamma‐delta T‐cell clones with a survival advantage are selected through a multistep process, resulting in malignant transformation of lymphocyte subsets.^24^ There is conflicting data behind the degree of influence of TNF‐alpha inhibitors in the development of HSTCL. Factors such as chronic antigenic stimulation and genetic predisposition may play a more vital role in the pathogenesis of HSTCL in patients with immunodysregulatory disorders.^2^, ^4^, ^25^

The most frequent symptoms patients present with are B symptoms.^4^, ^7^, ^9^ The most common physical examination finding is splenomegaly.^4^, ^7^, ^9^ Hepatomegaly is reported in 40%‐88% of patients and lymphadenopathy is reported in up to 25% of patients.^4^, ^7^ Lymphadenopathy is uncommon, reported in less than 25% of patients.^7^ The most frequent laboratory abnormalities are cytopenias.^2^, ^4^, ^7^ Splenic sequestration may be a cause of the cytopenias; however, the severity of cytopenias tends to correlate with disease progression, even in patients who undergo a splenectomy. Hence the pathogenesis of cytopenias is unclear.^2^, ^4^, ^9^ Myelodysplasia has also been suggested as an etiology of the cytopenias; however, morphologic characteristics of dysplasia have not been found to correlate with the presence of severity of cytopenias.^2^ Less than 10% of patients present with a lymphocytosis.^2^ About 90% of patients present with an elevated beta‐2 microglobulin level, 50%‐60% of patients present with an elevated serum lactate dehydrogenase, and about 50% of patients have a transaminitis at diagnosis.^7^

Our patient was also diagnosed with HLH, thought to be secondary to the lymphoma. HLH is a well‐recognized but rare entity that occurs in HSTCL. The reported incidence of HLH with a concurrent diagnosis of HSTCL is 5%.^7^ HLH is an inflammatory disorder driven by an unregulated activation of macrophages and lymphocytes.^10^ HLH tends to have a pediatric and young adult predominance.^10^ It is characterized by constitutional symptoms, splenomegaly, and cytopenias. HLH can be triggered by numerous conditions including viral infections, most commonly Epstein Barr virus (EBV), malignancies, and autoimmune disorders.^10^ Given the nonspecific symptoms of HLH there is often a delay in diagnosis. It remains vital that when a diagnosis of HLH is made in an adult, providers must investigate an underlying triggering disorder.

The prognosis of HSGDTL is dismal, with median survival less than one year from diagnosis.^2^, ^4^ Poor prognosticators include an elevated serum bilirubin (>1.5 mg/dL), elevated serum LDH, and trisomy 8 at time of diagnosis.^7^ The presence of i(7q) or aberrations of chromosome 7 may also confer a poor prognosis however larger studies are required to confirm this statistically insignificant finding.^7^ One group demonstrated that only INO80 gene mutation showed a tendency toward improved survival and alternative gene mutations including STAT3 and STAT5B did not draw a parallel with survival.^19^ Other clinical, pathological, and laboratory factors studies in HSGDTL have not demonstrated any prognostic significance.^2^, ^7^

The refractory and rare nature of HSTCL poses an extreme challenge in treatment. There is no established standard treatment. Most studies in literature have excluded patients with HSTCL due to its rarity, hence the paucity of data. The median overall survival (OS) ranges 3‐28 months with chemotherapy.^7^, ^9^ Two thirds of patients have a response to anthracycline‐based therapy however complete remission (CR) is uncommon.^7^, ^9^ Salvage treatments include alemtuzumab, antigamma delta T‐cell receptor monoclonal antibodies, and anti‐CD44 therapy.^2^, ^7^, ^9^ There are a handful of reports that suggest the addition of alemtuzumab and/or cladribine to anthracycline‐based regimens (classically CHOP) as first‐line chemotherapy may be of benefit, however this tactic needs further study.^26^

The use of hematopoietic stem cell transplant (HCT) in patients with HSTCL has been of interest lately. A study by the European Bone Marrow Transplant (EBMT) Lymphoma Working Party included 25 patients with HSTCL who received a HCT and demonstrated a median survival of 36 months.^27^ Of the 18 patients who received an allogeneic HCT (allo HCT), 2 eventually relapsed and of the 7 patients who received an autologous HCT (auto HCT), 5 eventually relapsed and died.^27^ The investigators implied that the transplant benefit relies on the graft‐versus‐lymphoma (GVL) effect conferred by an allo HCT, conferring a long‐term survival for some patients with HSGDTL.^27^ A systematic review of reports of allo HCT in 44 HSTCL patients demonstrated that 35% of the patients relapsed, all of which occurred within 1.5 years post transplant and no patients relapsed after 1.5 years.^28^ Again, the investigators suggested that the GVL effect presented by allo HCT can provide a long‐term survival benefit in some HSTCL patients.^28^ Yabe et al^7^ evaluated 12 patients with HSTCL who received HCTs at MD Anderson Cancer Center, and although the data was not statistically significant, it did suggest that long‐term survival was feasible with allo HCT.

Unfortunately our patient did not have genetic studies performed. JAK1/2, STAT3, STAT5B, and PI3KCD have been identified as potential therapeutic targets by genetic studies.^29^ There are ongoing studies utilizing chimeric antigen receptor (CAR) T‐cell based immunotherapy in relapsed or refractory HSGDTL.

## 4 CONCLUSION

Our case emphasizes the significance of investigating an underlying trigger such as lymphoma for secondary HLH. Due to its rarity and non‐specific signs and symptoms, a delay in diagnosis of HSGDTL may occur. Recognition of this T‐cell lymphoma is paramount in order to manage patients aggressively with systemic chemotherapy and consider early HCT. Furthermore, illustration of more cases with new biological features is required with a goal of developing novel therapeutic agents in the future. This field is in dire need of further research in the pathophysiology of the HSGDTL and treatment strategies.

## CONFLICT OF INTEREST

None declared.

## AUTHOR CONTRIBUTION

PHB: the first author, conceptualized the case report, collected the data included under Case Presentation and conducted a thorough literature review and drafted the manuscript. LTR: the second (corresponding) author collected the images included under Case Presentation, conducted a literature review and drafted and revised the manuscript with the other two co‐authors. SSA: the third author supervised the conceptualization of the case report, helped acquire data and critically revised the manuscript and provided final approval for its publication.

## References

[1] Yabe M , Miranda RN , Medeiros LJ . Hepatosplenic T‐cell lymphoma: a review of clinicopathologic features, pathogenesis, and prognostic factors. Hum Pathol. 2018;74:5‐16. 29337025 10.1016/j.humpath.2018.01.005

[2] Ferreri A , Govi S , Pileri SA . Hepatosplenic gamma‐delta T‐cell lymphoma. Crit Rev Oncol Hematol. 2012;83:283‐292. 22047938 10.1016/j.critrevonc.2011.10.001

[3] Gaulard P , Jaffe ES , Krenacs L , Macon WR . Hepatosplenic T‐cell lymphoma In: Swerdlow SH, Campo E, Harris NL, Jaffe ES, Pileri SA, Stein H, Thiele J, eds. WHO Classification of tumours of haematopoietic and lymphoid tissues. Lyon: IARC; 2017:381‐382.

[4] Falchook GS , Vega F , Dang NH , et al. Hepatosplenic gamma‐delta T‐cell lymphoma: clinicopathological features and treatment. Ann Oncol. 2009;20:1080‐1085. 19237479 10.1093/annonc/mdn751 PMC4092251

[5] Yabe M , Medeiros LJ , Daneshbod Y , et al. Hepatosplenic T‐cell lymphoma arising in patients with immunodysregulatory disorders: A study of 7 patients who did not receive TNF‐α inhibitor therapy and literature review. Ann Diagn Pathol. 2017;26:16‐22. 28038706 10.1016/j.anndiagpath.2016.10.005 PMC5560101

[6] Thai A , Prindiville T . Hepatosplenic T‐cell lymphoma and inflammatory bowel disease. J Crohns Colitis. 2010;4:511‐522. 21122554 10.1016/j.crohns.2010.05.006

[7] Yabe M , Medeiros LJ , Tang G , et al. Prognostic factors of hepatosplenic T‐cell lymphoma (HSTCL): A clinicopathologic, immunophenotypic, and cytogenetic analysis of 28 patients. Am J Surg Pathol. 2016;40:676‐688. 26872013 10.1097/PAS.0000000000000614

[8] Durani U , Go RS . Incidence, clinical findings, and survival of hepatosplenic T‐cell lymphoma in the United States. Am J Hematol. 2017;92:E99‐E101. 28263402 10.1002/ajh.24711

[9] Belhadj K , Reyes F , Farcet JP , et al. Hepatosplenic gammadelta T‐cell lymphoma is a rare clinicopathologic entity with poor outcome: report on a series of 21 patients. Blood. 2003;102:4261‐4269. 12907441 10.1182/blood-2003-05-1675

[10] Jordan MB , Allen CE , Weitzman S , et al. How I treat hemophagocytic lymphohistiocytosis. Blood. 2011;118(15):4041‐4052. 21828139 10.1182/blood-2011-03-278127 PMC3204727

[11] Vega F , Medeiros LJ , Bueso‐Ramos C , et al. Hepatosplenic gamma/delta T‐cell lymphoma in bone marrow. A sinusoidal neoplasm with blastic cytologic features. Am J Clin Pathol. 2001;116:410‐419. 11554170 10.1309/BM40-YM6J-9T3X-MH8H

[12] Weidmann E . Hepatosplenic T cell lymphoma. A review on 45 cases since the first report describing the disease as a distinct lymphoma entity in 1990. Leukemia. 2000;14:991‐997. 10865963 10.1038/sj.leu.2401784

[13] Macon WR , Levy NB , Kurtin PJ , et al. Hepatosplenic alphabeta T‐cell lymphomas: a report of 14 cases and comparison with hepatosplenic gammadelta T‐cell lymphomas. Am J Surg Pathol. 2001;25:285‐296. 11224598 10.1097/00000478-200103000-00002

[14] Wang CC , Tien HF , Lin MT , et al. Consistent presence of isochromosome 7q in hepatosplenic T gamma/delta lymphoma: a new cytogenetic‐clinicopathologic entity. Genes Chromosomes Cancer. 1995;12:161‐164. 7536454 10.1002/gcc.2870120302

[15] Przybylski GK , Wu H , Macon WR , et al. Hepatosplenic and subcutaneous panniculitis‐like gamma/delta T cell lymphomas are derived from different Vdelta subsets of gamma/delta T lymphocytes. J Mol Diagn. 2000;2000(2):11‐21. 10.1016/s1525-1578(10)60610-1 PMC1906890 11272897

[16] Navarro JT , Ribera JM , Mate JL , et al. Hepatosplenic T‐gammadelta lymphoma in a patient with Crohn's disease treated with azathioprine. Leuk Lymphoma. 2003;44(3):531‐533. 12688327 10.1080/1042819021000035662

[17] Mackey AC , Green L , Liang L , Dinndorf P , Avigan M . Hepatosplenic T cell lymphoma associated with infliximab use in young patients treated for inflammatory bowel disease. J Pediatr Gastroenterol Nutr. 2007;44(2):265‐267. 17255842 10.1097/MPG.0b013e31802f6424

[18] Nicolae A , Xi L , Pittaluga S , et al. Frequent STAT5B mutations in gam‐ madelta hepatosplenic T‐cell lymphomas. Leukemia. 2014;28:2244‐2248. 24947020 10.1038/leu.2014.200 PMC7701980

[19] McKinney M , Moffitt AB , Gaulard P , et al. The genetic basis of hepatosplenic T cell lymphoma. Cancer Discov. 2017;4:369‐379. 10.1158/2159-8290.CD-16-0330 PMC5402251 28122867

[20] Alonsozana EL , Stamberg J , Kumar D , et al. Isochromosome7q:the primary cytogenetic abnormality in hepatosplenic gammadelta T cell lymphoma. Leukemia. 1997;11:1367‐1372. 9264394 10.1038/sj.leu.2400742

[21] Herrinton LJ , Liu L , Weng X , Lewis JD , Hutfless S , Allison JE . Role of thiopurine and anti‐TNF therapy in lymphoma in inflammatory bowel disease. Am J Gastroenterol. 2011;106:2146‐2153. 22031357 10.1038/ajg.2011.283

[22] Giacomelli R , Parzanese I , Frieri G , et al. Increase of circulating gamma/ delta T lymphocytes in the peripheral blood of patients affected by active inflammatory bowel disease. Clin Exp Immunol. 1994;98:83‐88. 7923890 10.1111/j.1365-2249.1994.tb06611.x PMC1534185

[23] Soderstrom K , Bucht A , Halapi E , et al. Increased frequency of abnormal gamma delta T cells in blood of patients with inflammatory bowel diseases. J Immunol. 1996;156:2331‐2339. 8690925

[24] Sokol H , Beaugerie L . Inflammatory bowel disease and lymphoproliferative disorders: the dust is starting to settle. Gut. 2009;58:1427‐1436. 19749141 10.1136/gut.2009.181982

[25] Kelsen J , Dige A , Schwindt H , et al. Infliximab induces clonal expansion of gammadelta‐T cells in Crohn's disease: a predictor of lymphoma risk? PLoS ONE. 2011;6:e17890. 21483853 10.1371/journal.pone.0017890 PMC3069033

[26] Jaeger G , Bauer F , Brezinschek R , Beham‐Schmid C , Mannhalter C , Neumeister P . Hepatosplenic gammadelta T‐cell lymphoma successfully treated with a combination of alemtuzumab and cladribine. Ann Oncol. 2008;19:1025‐1026. 18375525 10.1093/annonc/mdn119

[27] Tanase A , Schmitz N , Stein H , et al. Allogeneic and autologous stem cell transplantation for hepatosplenic T‐cell lymphoma: a retrospective study of the EBMT Lymphoma Working Party. Leukemia. 2015;29:686‐688. 25234166 10.1038/leu.2014.280

[28] Rashidi A , Cashen AF . Outcomes of allogeneic stem cell transplantation in hepatosplenic T‐cell lymphoma. Blood Cancer J. 2015;5:e318. 26047388 10.1038/bcj.2015.43 PMC4648481

[29] Travert M , Huang Y , de Leval L , et al. Molecular features of hepatosplenic T‐cell lymphoma unravels potential novel therapeutic targets. Blood. 2012;119:5795‐5806. 22510872 10.1182/blood-2011-12-396150 PMC3779008

